# Biological Practices and Fields, Missing Pieces of the Biomimetics’ Methodological Puzzle

**DOI:** 10.3390/biomimetics5040062

**Published:** 2020-11-18

**Authors:** Eliot Graeff, Nicolas Maranzana, Améziane Aoussat

**Affiliations:** Laboratory of Product Design and Innovation, LCPI, Arts et Metiers Institute of Technology HESAM University, 151 Bd de l’Hôpital, 75013 Paris, France; nicolas.maranzana@ensam.eu (N.M.); ameziane.aoussat@ensam.eu (A.A.)

**Keywords:** biomimetics, biomimicry, biological tools, pluridisciplinarity, innovation

## Abstract

Facing current biomimetics impediments, recent studies have supported the integration within biomimetic teams of a new actor having biological knowledge and know-how. This actor is referred to as the “biomimetician” in this article. However, whereas biology is often considered a homogenous whole in the methodological literature targeting biomimetics, it actually gathers fundamentally different fields. Each of these fields is structured around specific practices, tools, and reasoning. Based on this observation, we wondered which knowledge and know-how, and so biological fields, should characterize biomimeticians. Following the design research methodology, this article thus investigates the operational integration of two biological fields, namely ecology and phylogenetics, as a starting point in the establishment of the biomimetician’s biological tools and practices. After a descriptive phase identifying specific needs and potential conceptual bridges, we presented various ways of applying biological expertise during biomimetic processes in the prescriptive phase of the study. Finally, we discussed current limitations and future research axes.

## 1. Introduction

This introduction contextualizes our work before it describes a state of the art on the current methodological limitations, leading to the research question of this article.

### 1.1. Positioning of the Article

Biomimetics is defined as “the interdisciplinary cooperation of biology and technology or other fields of innovation with the goal of solving practical problems through the function analysis of biological systems, their abstraction into models, and the transfer into and application of these models to the solution” [1].

Over the past decades, biomimetics has established itself as one of the most promising strategies to support innovative [2] and environment-friendly products [3]. However, it is still at a development and structuration phase, and because of the intrinsic difficulty of combining biology and engineering, its implementation in the industrial world remains highly marginal [4]. Where engineering-centered steps [5] are well-known by engineers, and so well performed by design teams, steps centered on biological information (biology-centered steps) are entirely new and lack efficiency [6]. If we take engineering-centered steps as a point of comparison, we can identify three main aspects that enable their performance: defined fields of expertise (mechanical engineering, product design, etc.), defined actors (mechanical engineers, product designers, etc.) and a defined methodological framework. With biology-centered steps, none of those aspects are well-defined. Instead of determining which fields and which actors should perform these entirely new and highly specific steps, current approaches deal with the provision and analysis of biological data to classic design team members (engineers, designers, ergonomists, etc.). Consequently, biological fields, practices and reasonings remain out of the biomimetic methodological scope. If biology is the subject of those steps, and biological data are used as a resource, biology isn’t considered as the engine of resolution.

Based on that logic, numerous articles have been published to support the integration of biologists within biomimetic design teams [7,8,9,10]. Wondering how, when, and for what purposes biologists should be integrated, these studies quickly underlined that the community wasn’t only referring to existing biologists, but also pointed to the need for a new profile, leading to the question of its definition.

Thanks to a pluridisciplinary team combining backgrounds in biology, biomimetics, and engineering design, and through exchanges with researchers of both communities, this article focuses on how to characterize this new actor’s biological knowledge, know-how, and tools.

Since each biological field encompasses a specific set of knowledge, know-how, and tools, this article hypothesizes that they are key elements to manipulate. Thus, through the formalization of conceptual bridges between biological fields and biology-centered steps, the hypothesis supports the idea that required knowledge, know-how, reasoning, and tools can be identified to better define the new team member having a background in biology.

In this respect, and based on the literature [11], ecology and phylogenetics are identified as key biological fields to integrate. Several characteristics of interest can then be underlined, they both (1) study all types of living beings (plants, animals, bacteria, fungi, etc.), (2) gather species into structured groups (in biocenosis or taxon), (3) reason at the level of organisms (as representants of species) allowing a combination of their respective concepts or reasonings, and (4) have a fundamental link with biological evolution. Through the presentation of these fields, their biological objectives, reasoning axes and tools, we managed to bring out conceptual bridges leading to practical applications. The impact of these contributions on the biomimetic process and on the definition of the new profile to integrate are discussed.

The final goal of this work is to point out a new area of research targeting the use of specific biological expertise in biomimetic projects. The work presented in this article only focuses on some of the numerous potential biological fields that could be integrated, raising exciting perspectives on biomimetic actors, practices, and a methodological framework.

### 1.2. State of the Art

This state of the art first presents the current scientific reflections regarding biomimetic practices and brings out the question of biological biomimetic practices through the prism of potential biological fields of expertise.

#### 1.2.1. Biomimetics Methodological Framework

Biomimetic processes follow two types of approach. Either they are initiated by biological discoveries, the biology push approach [1], or biomimetics is used as a problem-solving process, the technology pull approach [1]. Because of the potential industrial applications, the problem-solving reasoning has been the main target of engineering design research so far. Indeed, as for any innovative design theory, biomimetics first needed a methodological framework to turn a theoretical idea, inspired by living beings, into a highly complex reality.

**Figure 1:**
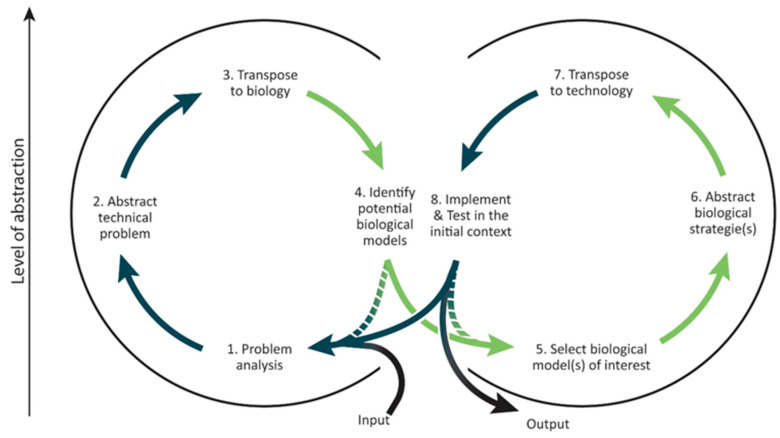
The unified problem-driven biomimetic process from Fayemi [5].

Thus, a great number of processes have been designed to help implement a technology pull approach, e.g., the procedural model of doing bionics [12], biomimetic design methodology [13], problem-driven analogical process [14], etc. Through a comparative analysis of these processes, Fayemi synthesized eight main steps representing an overall description of the technology pull biomimetic process [15]. Figure 1 presents the resulting process that will be considered as the process of reference in this article.

Along with these studies on biomimetic processes, the need for new tools soon emerged. Indeed, the lack of biological knowledge led engineers to face limitations during these processes. For instance, how to find, extract, and transfer biological models. Numerous tools were designed to deal with these issues [16], leading to the generation of tool-based processes, e.g., idea-inspire process [17] and biologically inspired concept generation [18], or to the improvement of existing ones.

Tools used in biomimetics have mainly three origins [19]: engineering, like the five whys [20] or technical contradictions [21]; biology, like 16 patterns of nature [22] or functional modelization [23], or are specifically designed for biomimetic purposes, like SAPPhIRE (State, Action, Part, Phenomenon, Input, oRgan, Effect) [17] or BIOTRIZ [24]. It must be specified that the tools that are said to come from biology, are neither biological tools nor tools designed to be used by biologists. They mainly allow engineers to learn about biological concepts and finding without considering inputs or perspectives from biology.

Despite these clear methodological progresses, some practical impediments still prevent biomimetics to reach its full potential.

#### 1.2.2. Practical Impediments and Current Resolution Strategies

Three main obstacles regarding the use of biological data during the practice of biomimetics have been identified [6,25]. First, the findability of biological models, corresponding to the team’s ability to find biological models matching their abstracted technological problem during the 4th step of the process of reference above mentioned (Figure 1). This difficulty is multifactorial, since design teams need to identify what to search for, where to search, and how to search.

- What to search for? Overall, teams are asked to search for biological models. But again, what is a biological model in biomimetics? Is it a biological process? An organism? A biological system? This ill-define “biological model” represents a first difficulty.
- Where to search for? Most of the scientific findings are stored online on various types of platforms (scientific databases, popular science website, etc.). Facing these various possibilities, the question of where to search, depending on the teams’ expertise and objectives, appears crucial.
- Finally comes the “how”, namely how to identify the proper keywords leading to the searched information?

Secondly is the recognizability issue. It represents the difficulty of design teams to judge the relevancy of a model. Mainly associated with the 5th step of the process, recognizability is also a key factor during the 4th step of the process. The association of an efficient search and a relevant set of choices, or judgement, (on keywords, on articles, etc.) may lead to a successful 4th step:

- How to identify selection criteria and perform evaluation on those criteria to sort the various models that are considered relevant?
- How to assess the quality of the biological data, whether it is enough to formulate a biological model, and its adequacy?

Finally, the third and most cross-cutting issue, is the understandability of biological data. It represents the ability of the team to interpret biological data into coherent information. It has a crucial impact during the abstraction of the biological data in the 6th step but appears fundamental in all biology-centered steps. Without knowledge in biology, relevant information, websites, keywords, or requests can be extremely hard to identify. Without a clear understanding of the data, choices made during the search and selection step can be based on irrelevant criteria, on fixations, or just on having a “good feeling”, which may lead the team to make some mistakes during the design process.

Where we focus on those three impediments in this article, various other axes can be considered at different phases of biomimetic projects, such as the technological feasibility [26] or the industrial-scale production and profitability of biomimetic product [27].

So far, three main resolution strategies have been considered to deal with biological data, databases of structured models [28,29,30], natural language web search [31,32,33] along with artificial intelligence (AI) [33,34] and finally approaches based on highly abstracted principles [35,36]. Each strategy has advantages and drawbacks that are synthesized in Table 1 for clarity.

Regardless of their types, all these solutions have been designed from an engineering standpoint. None of these approaches use biology and its associated reasonings, practices, or tools as resolution engines. As a result, biology as a discipline and a science remains beyond the scope of these strategies. The next section will then present the theoretical and practical links existing between biology and biomimetics to underline the interest of considering the integration of biology as an alternative approach.

#### 1.2.3. A Biological Approach, another Alternative

More and more biological concepts are used to optimize current biomimetic tools. First, the concept of “environment” has been studied because it is well understood by design teams which see in this concept a synonym of “context” or “super-system”. They can look at it, experience it, and are easily convinced of its interest. The global tendency of supporting a more systemic, more holistic, approach of design also led engineers to be more comfortable integrating such concepts. Concretely, it is mainly used to generate keywords in tools such as BIOScrabble [44,45], represent and compare biological models like in the four-boxes model [46] or in Badarnah’s pinnacles analyzing matrix [47] or the adaptation of the functional causal model SAPPhIRE [17,48]. Yen et al. advise to consider extreme environments to find “high-performing biological systems” [10], also called “champion adapters” by Helms [49]. Kennedy et al. [50] worked on biological models’ search and identified four frames of inquiries: “similar context” and “extremes”, based on environmental consideration and “convergence” and “stasis”, associated with evolutionary concepts.

Moreover, various aspects have been identified to specifically characterize biological systems [51,52,53,54]. Those concepts (e.g., multiple systemic scales, multifunctionality, etc.) have been used to support the formalization [55,56], comparison, and selection [51] of biological systems through specific models. It also led to the formalization of design guidelines such as “life principles” [57,58], and can even be directly integrated within approaches using abstracted biological strategies through the concept of trade-offs [43] at the origins of the ontological approaches (Section 1.2.3) [6,36].

Finally, the identification of biological concepts of interest enables the consideration of biological fields through extracted elements perceived as adjustment variables within algorithms and Artificial Intelligence. Thus, Kim et al. [48] considered taxonomic classification tool DELTA [59,60] along with ecological parameters (geography, resources and phylogeny) [61] as the source of criteria for search and association tools. Considering actual biological classifications in the design of computational approaches represent a real step forward in the integration of biology.

Where tools’ optimization is of great interest, one can argue that it only represents one side of the coin. The “interdisciplinary cooperation of biology and technology” calls for a broader synergy that might be reached through the integration and adaptation of biological practices. In the end it all comes down to expertise. Most current design team members have a limited knowledge and know-how in terms of biology and so, logically, will use tools and strategies they are comfortable with and trained for.

In an article discussing the oversimplification of considering biological evolution as a blind justification for biomimetics, Fish underlined various key aspects to consider and limitations to take into account [54]. More specifically, he pointed out key differences between biological and technological evolutionary processes (no conscious evolution, error as a key parameter, etc.) and drew attention on the danger of using shortcuts to generate certainties (organisms are not optimized, life doesn’t lead to perfect solutions [11], etc.). Natural selection thus is a highly complex process which relies on multifactorial constraints (see Section 3.1.2) leading to the adaptation of multifunctional traits, as stated by Vincent about biological material, “many materials have more than one function” [62].

In her studies on BID (Bio-Inspired Design) pedagogy, Yen also reminds that “evolution is a historical process” [63] with reference to Darwin theory of natural selection which postulates that evolution is mainly gradual [64], meaning that it is constrained by evolutionary history, traits are adapted from existing ones. She also underlined the difficulty of finding an adequate level of abstraction for biological models, since at some level the all share the same “ultimate evolutionary objective, to survive, which is so universal that it gives no additional guidance” [10].

Altogether these observations represent fundamental pitfalls for practitioners wanting to use biology without proper training during biomimetic projects. One solution that quickly appeared in the literature is the cooperation of actors from both biology and technology. More recently, the integration of a new team member, trained in biology for biomimetics purposes, also emerged and is discussed in the next section.

#### 1.2.4. Integration of Stakeholders with a Background in Biology

Recent studies have investigated such research axis, to legitimate the integration of stakeholders from biology through quantitative and qualitative studies [8,9,65], to point out the need for new communication support [66] and to properly define the actual needs and expectation of design teams towards biologists [10,67].

The design of specifications surrounding this interdisciplinary collaboration led to the characterization of two different profiles to consider. A first one having a vertical, specific and deep biological knowledge, mainly embodied by researchers in biology, and a new profile having a horizontal and broad biological knowledge specifically adapted for biomimetics, which is not currently formalized and so do not properly exist yet [51,68].

The integration of biological researchers appears of great interest specifically during the abstraction of biological strategies (step 6) which requires deep knowledge on the strategy [69] before the rise of the abstraction level can occur [68]. Their practice in biomimetics is then well-defined since their role is essentially the same one than their usual jobs, explaining how does a biological model work. Still, small but crucial differences can be underlined. First, they will have to make their knowledge available to design teams, whose biological knowledge is often low. Secondly, they might look at their studies from a new prism since the questions emerging from biomimetic needs can be different from the ones faced in biological research. Finally, where their role is rather determined, their incentives aren’t and remain a subject to address.

The integration of horizontal biologists is recognized to be of great potential all along the process but more specifically during the biology-centered steps (steps 3–5) [68]. During those steps, they may help design teams to enter biology, explore the biological diversity, select pertinent models and properly transfer this information to the rest of the team [7,8,10,70]. However, even if they are described in terms of potential contributions, objectives, and involvement steps, the literature doesn’t properly prescribe how will they be able to reach those objectives. For example, Yen stated that “advanced students (in biology) may even learn to find relevant examples based on phylogenetic relatedness” [10], already pointing out the potentialities of phylogenetics presented in this article but without further information. Therefore, they remain ill-defined regarding their practice, skills, and field of expertise. The main reason explaining that situation is that steps 3–5 have no direct equivalent in biology. Thus, conceptual bridges need to be formalized to allow a proper adaptation of biological practices, tools, and reasoning to biomimetics.

**Figure 2:**
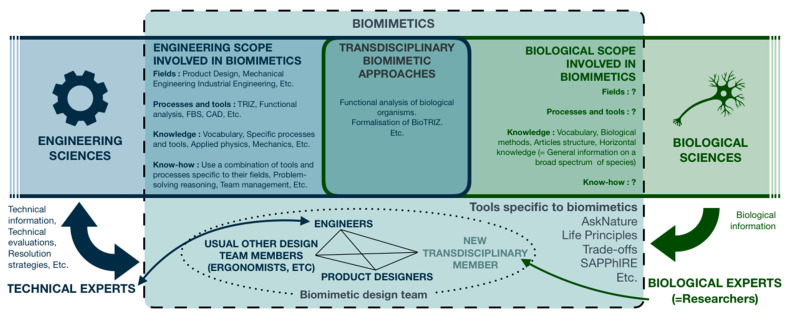
Diagram representing a summary of the currently identified fields, methods, tools and actors involved in biomimetics.

Facing the observation made in this section, determining which part of biology, and so which biological fields, to integrate in the context of biomimetics would naturally bring answers on the characterization of this new profile’s know-how, knowledge, practices, and tools (Figure 2).

This state of the art on biomimetics’ methodological framework and practical impediments lead us to consider the integration of new team member trained in biology as a relevant solution. The next section will then synthesize the observation made in the state of the art to formalize the research question addressed in this article.

### 1.3. Synthesis and Research Question

From the observations presented in the state of the art, we have pointed out that despite great methodological progress, biomimetic practice still faces biology-related impediments. Various approaches have been designed to solve these issues and their advantages and drawbacks have been summarized in Table 1.

However, as previously pointed-out, the current situation is rather paradoxical since mainly engineering-centered answers are designed to solve biology-centered problems. The integration of biological practices is then considered in this article as an alternative solution. So far, those new practitioners were referred to has horizontal biologists because of their broad biological knowledge but since these profiles need to be specialized in biomimetics, the term “biologists” appears reductive. This new team member therefore needs a specific name and, as such, will be referred to as a “biomimetician”.

This article then deals with the following research question: what are biomimeticians’ specific biological knowledge, know-how, and tools?

The hypothesis of this article presents a partial answer through the formalization of skills based on two biological fields, namely ecology and phylogenetics, as the first key fields of interest for biomimeticians to face above-mentioned impediments. Focusing on the findability issue, this article explores those two fields and various concepts, reasoning or tools that can be integrated through a biological expertise, are thus presented. The needs emerging from the adaptation of biological practice to biomimetic practice, and the fact that numerous biological fields remain to include, are underlined, raising significant research perspectives. Potential impacts of the integration of those first two biological fields on the biomimetic process are then discussed.

At an overall scale, we want to approach the practice of biomimetics with the standpoint of practitioners having a background in biology and underline their potential specific contributions.

## 2. Material and Methods

This section presents the guiding themes of this article and the approach we chose to structure our reasoning. The aim of our global study is to formalize the use of biological fields during biomimetic practices, starting with biological-centered steps. This article mainly deals with the two first biology-centered steps, steps 3 and 4.

To make our reasoning as clear as possible, we used the first three steps of the design research methodology [71] to structure our exploratory work on these two steps:

1. Research clarification: This phase corresponds to the Section 1.
2. Descriptive phase: Based on the literature, we pointed out specific impediments on the targeted steps. This phase is divided between Section 3.1.1 (for the 3rd step) and Section 3.2.1 (for the 4th step).
3. Prescriptive phase: We exposed conceptual bridges linking those pitfalls with biological fields and prescribed practical contributions. We also underlined the potential impact on the process to anticipate and better integrate these new practices. This phase is divided between Section 3.1.2 (for the 3rd step) and Section 3.2.2 (for the 4th step).

Finally, we described the impact of our contributions on the characterization of the biomimetician’s expertise (Section 3.3 and discussed the limitations and numerous perspectives of our work (Section 4).

## 3. Results

### 3.1. Findability and the Ill-Defined “Transposition to Biology”

#### 3.1.1. First Descriptive Step: the 3rd Step, the Initial Grain of Sand

If we study the different steps of the biomimetic cognitive reasoning, practitioners need to get from abstracted design issues (end of step 2) to transposed biological issues (end of step 3), to the identification of biological models (step 4). As this identification is based on the output generated during the 3rd step, it seems logical to look at this step before going any further.

In the problem-driven unified process formalized by Fayemi (Figure 1), the 3rd step was explained in those few lines: “the establishment of a generic model combined with the identification of the functions of interest makes possible the transposition of the problem and its environment to biology. At this step, practitioners usually formulate a request. The purpose of this request is to explore Nature’s ways to perform one or several functions. This 3rd step is crucial since the overall biomimetic results is deeply impacted by the request formulation.” (from Fayemi [19] translated by the authors). In the above-mentioned definition, three main aspects emerge:

- The required inputs from the second step: generic models and associated functions of interest.
- The goal of the 3rd step: to transpose technical problems and their environments to biology.
- The form of the 3rd step’s outputs: requests to be used as bridges between the abstracted model and the biological solutions.

However, in practice, this theoretical ground can be overtaken by the tools used for the 4th step. For example, if a team usually performs the 4th step with IDEA-INSPIRE than they will use the SAPPhIRE formalization during the 2nd and 3rd step, those who use AskNature will design requests based on the Biomimicry Taxonomy framework, etc.

A fundamental shift then emerges. The goal of the 3rd step doesn’t appear as the “transposition of the problem and its environment to biology” but as the formalization of problems into a format fitting the requirement of the 4th step’s tools. The notion of biology fades away as practitioners struggle to apprehend the biological world and so rely on tools made by and for engineers (Section 1.2.3). Even with thesaurus and translation tools, this transposition step may shift from reflected decisions made thanks to a change of conceptual and cognitive framework, to the direct consequences of semantics association made through the tools.

As a result, we argue that this theoretical drift between the initial goal and the practical implementation may represent a key lever to consider. One reason behind this drift, might be the concept of transposition that isn’t unambiguous and applicable by itself. Whereas some will hear translate, others will hear apply or adapt, and most people may have trouble performing the action even if they understand its sense. As an example, the Cambridge Dictionary defines transposition as “the act or process of changing something from one position to another, or of exchanging the positions of two things”. Applying this definition in the context of transposition to biology isn’t an easy task. Consequently, practitioners interpret this step without a real coherence regarding the initial goal of the 3rd step, but rather with the obvious need of fitting the 4th step’s requirements.

The second reason that may explain this drift derived from the first. Since transposition, as well as biological concepts, aren’t clear for design teams, practitioners may fall back on their design habits, leading to a bias of fixation effect on the traditional engineering process. Hence, requests are formalized to represent functional requirements through a mix of biological, “biologized” and engineering concepts, while keeping an engineering standpoint. Practitioners lack knowledge and skills regarding biology and so the door on the biological world often remains closed.

#### 3.1.2. Prescriptive Step: Ecology and Solution Spaces

More than only an interface between engineering and biology, the goal of this 3rd step is to define, within biology, the scope of the search occurring during the 4th step. Thus, the 3rd step’s goal is to perform an analogical reasoning based on problems not directly on solutions, in the words of Fu, to generate “innovative design problem formulation” [72].

As a result, we propose to rename the 3rd step “project problems into biology to identify solution spaces”. If various other studies have proposed semantics to represent this step, like “reframe”, “biologize” the problem [49] or “problem definition phase” [13], this new reformulation clarifies the activity to perform, “project problems”, but also specifies the scientific field to reach, “biology”, and the expected output, “solution spaces”.

Facing this new definition, the underlying cognitive process can also be better understood. The 3rd step is thus performed through the projection of abstracted problems into biology to identified biological embodiments of the abstracted problems. These embodiments are then mapping problem spaces within biology. Thus, in those spaces, biological organisms are facing problems analogous to the ones faced in our technical model. Taking the side of these organisms, problem spaces represent solution spaces. The choice of using the concept of solution space over problem space is to focus on the idea that those spaces are encompassing numerous, sometimes highly diverse, solutions for a given combination of problems. The 4th step will then lead biomimetic teams to search and identify models within these solution spaces. Solution spaces thus form a map delimiting the scopes of high potentialities from those that may be less relevant.

Along with this conceptual formalization, it also appears crucial for solution spaces to be characterized through specific criteria (= keywords) along with their associated conceptual network. These criteria will then allow design teams to find pathways (= requests) and to manipulate solution spaces to identify the biological models they contained. The interest of this formalization is that it should be perfectly compatible with the current approaches while also allowing the team to switch toward a more biological standpoint.

In current practices, biomimetic practitioners are used to focus on functions, and so solution spaces appear functional (FSS). Functional problems are abstracted and projected to identify a space gathering biological models that perform a given function. Spaces are then characterized through functional keywords as described in the state of the art.

In biology, the space containing a given organism, and so all biological models it might involve, is called its environment. As presented in the state of the art, considering the environment for the generation of keywords is supported in the literature (Section 1.2.4), but again, from an engineering perspective, as a new tool to increase engineers’ ability to reach biology. In the case where a biomimetician is integrated in the team, biological tools, reasoning, and concepts may potentially be directly used as engines of resolution. Taking a biological standpoint, the field studying the “interactions among living things and their environment” is called ecology [73].

In ecology, ecosystem represents the association above described between a biotope (non-living elements like climatic context, soil chemistry, etc.), a biocenosis also called an ecological community (network of living beings associated with a biotope), and their interactions [74]. In order to underline the interest of these concepts in biomimetics, this sub-section presents, at an overall scale, the link between ecology and the biological evolutionary process.

The evolutionary process, engine of the emergence of biological solving strategies, is somewhat reminiscent of the double diamond process describing the design process of innovation [75]. For a given population, divergent phases lead to an increase of the genetic diversity, through various mechanisms such as independent assortment during sexual reproduction, crossing-over during meiosis or genetic mutation in germ cells, and convergent phases lead to the selection of organisms having specific genetic sequences. Over time, the proportion of the genetic material in the population evolves, leading to the adaptation of the population. It must be clearly underlined that, where organisms are selected, they however do not adapt in a genetic, evolutionary sense, only populations adapt in a dynamic way, as the resultant of numerous organisms’ selection.

Multiple blind mechanisms (not oriented by choices or will of any kind) explain this selection step. One of them, known as natural selection, is further developed in this section leaving aside other mechanisms such as genetic drift, population bottleneck, etc. This section only presented the overall mechanism of this complex biological phenomenon. Thus, for more information about evolutionary processes we refer the reader to the literature [76].

The main engine of natural selection is called evolutionary pressure and depends on factors impacting the ability of organisms to produce offspring. These factors can be biotic (have a biological source) or abiotic (have a non-biological source) and can have an impact on various parameter such as the organism’s ability to mate (selection of sexual traits for example), its fertility (the organism mates but doesn’t produce any offspring) or its ability to survive (limiting the time during which it will be able to reproduce) [77]. In the context of our study, we will focus on the last aspect, survival, to link evolution and adaptation to environmental constraints.

Overall, the chance of survival of an organism depends on the adequacy between its traits and its environment. This adequacy is referred to as fitness in biology [77]. To illustrate this process, let’s consider a fishing net as part of an environment. The organisms which size (selected trait) is bigger than the size of the mesh (environmental constraints) will be eliminated, and so can’t reproduce with other organisms remaining in the environment. On the contrary, organisms that are smaller than the mesh of the net aren’t sensitive to this environmental constraint and so survive and can have offspring. Environmental constraints combined with specifically inadequate traits thus lead a given organisms to their death, preventing them to reproduce and so to pass on their genes. Hence, in a rather stable environment, the fitness of the organisms composing the population increases through generations. This phenomenon, also known as the “survival of the fittest”, is one of the main mechanisms of natural selection [77].

This example illustrates the selection process through a single environmental constraint, the size of the mesh. Based on such a constraint, some traits are strongly selected, leaving little room for variability (like the size of the organism in our example), others are moderately selected (like the species of organisms in our example), and others aren’t correlated with a change in the survival ability, leading to a stable diversity (for example the various color patterns of fishes in our example).

However, it must be underlined that in nature, this mechanism simultaneously involves numerous constraints coming from both the biotope (abiotic constraints like temperature, humidity, mesh size of a net, etc.) and the biocenosis (biotic constraints like predation, sexual traits, etc.) of the ecosystems. For example, where we pointed out that color patterns aren’t a trait selected by a fishing net, it can be strongly selected as a sexual trait (impacting the mating probabilities) and so still lead to specific evolutionary paths. Moreover, since organisms live in a constraining environment, evolution has limits and selection thus appears to deeply rely on balance, explaining the approach based on trade-offs supported by Vincent [43]. This notion is crucial to understand, as stated by Yen, that evolution is a process towards “a good solution, as opposed to the best solution” as the result of “multiple, sometimes conflicting, demands or constraints” [63].

Based on the biological processes above mentioned, the development of a biological model of interest ultimately rely on environmental constraints of interest. However, in current biomimetic practices technical problems are mainly formulated with abstracted functions for the product to perform (see Section 1.2.2). While searching for biological models, practitioners then generate the following generic request: “Which organisms perform + a function?” leading them to identify functional solution spaces (FSS). The question that emerges is then: how to project abstracted technical problem (outputs of the 2nd step) into environmental spaces encompassing adapted biological organisms?

So far, articles of the literature that consider these environmental spaces to search, compare or select biological models approach environment as a new parameter to consider, without explaining how these spaces are determined from the design problem. Thus, when ESS are easy to determine, design teams might instinctively use ESS as presented in Yen or Kennedy’s studies (see Section 1.2.3). For example, if a team wonders “which biological entities perform the extraction of oxygen from water?” or “which biological entities resist high temperature?”, an aquatic habitat and a high temperature environment (deserts, submarine volcanos, hot water springs, etc.) respectively appear as clear ESS.

However, in other cases, ESS aren’t easily deductible and biomimetic teams then lack a systematic approach to project design problems to identify ESS. As an example, if practitioners wonder “which biological entities manage mechanical wear?” or “which biological entities optimize space/material?” ESS are less intuitive and might then be completely ignored during the search phase. Thus, we designed various guidelines in order to guide biomimeticians during the projection into biology.

Firstly, they have to realize that, from a biological perspective, a functional formulation focuses on solutions, not on problems [43]. For example, the extraction of oxygen from water is a solution to the problem of living under severe lack of oxygen because of an aquatic habitat. Consequently, extracting oxygen from water isn’t the only solution to this problem. Indeed, some evolutionary paths led organisms to deal with this constraint by periodically surfacing in order to breathe, like dolphins. Therefore, by focusing on the formulation of problems from the point of view of a living being, a cognitive shift can occur. A biomimetician may wonder “under which constraints does the extraction of oxygen from the environment become a strongly selected trait?” and so “what would be the problems leading to a biological adaptation towards oxygen extraction?”. Various characteristics defining solution spaces can then be identified. Among those, the oxygen concentration in the environment can be scarce, like in high altitude or in the soil, but oxygen can also be dissolved in a medium and so hardly accessible.

Thus, biomimetician should be able to identify environmental constraints for most design problem, resulting in a switch from functional paths to ecological paths of projection, “Which organisms (+ perform a function) + to deal with given environmental constraints?”.

Secondly, based on ESS, design teams should then be able to consider and combine both types of reasoning (functional and ecological) and so to increase the team’s ability to face the findability issue. As a result, a study targeting the decontaminate on of polluted water (abstracted into high concentrated fluid) in deep-sea facilities (abstracted into a parameter of high pressure) can reach biology through the projection into functional solution space “how does nature filter liquid?”, ecological solution space “which biological organisms survive in a high pressure, high concentrated fluid?” or a solution space combining these approaches, i.e., “which biological organisms filter high pressure, high concentrated fluids?”. Once a ESS is identified, previously published articles also offer guidelines to identified alternative ESS [10,50], such as spaces that represent reverse constraints and spaces that are only presenting one constraint pushed to an extreme (see Section 1.2.3).

Finally, more than only determining ESS, our work aims at considering ESS as inputs for biological tools and reasonings to be used by biomimeticians. To do so, ESS must match existing biological approaches. Various biological concepts can be used as ESS from an ecological standpoint. Depending on the studied environmental constraints, several classifications, such as the Köppen–Geiger classification [78] or the Holdridge life zones [79], have been designed to divide the earth into ecological spaces. Regardless of the chosen classification, the ideas developed below will apply.

In this article, one classification is taken as reference: the Olson classification better known under the name of WWF classification [80]. It has been chosen because it is known to be based not only on climatic parameters but also on parameters originating from biogeography. Biogeography is defined as “the study of the geographic distribution of plants and animals. It is concerned not only with habitation patterns but also with the factors responsible for variations in distribution.” [81].

Depending on the considered scale, this classification splits the earth into 20 ecozones (eight terrestrial and 12 marine), 28 biomes (14 terrestrial, seven marine and seven freshwater), and 1525 ecoregions (867 terrestrial, 232 marine and 426 freshwater). These various ecological concepts are defined as follows:

- Ecozones, “represent unique faunas and floras of different continents or ocean basins” [82].
- Biomes are described as “different areas of the world that share similar environmental conditions, habitat structure and patterns of biological complexity (e.g., beta diversity) and that contain communities with similar guild structures and species adaptation” [80].
- A terrestrial ecoregion is defined as “relatively large units of land containing a distinct assemblage of natural communities and species, with boundaries that approximate the original extent of natural communities prior to major land-use change.” [80].

**Figure 3:**
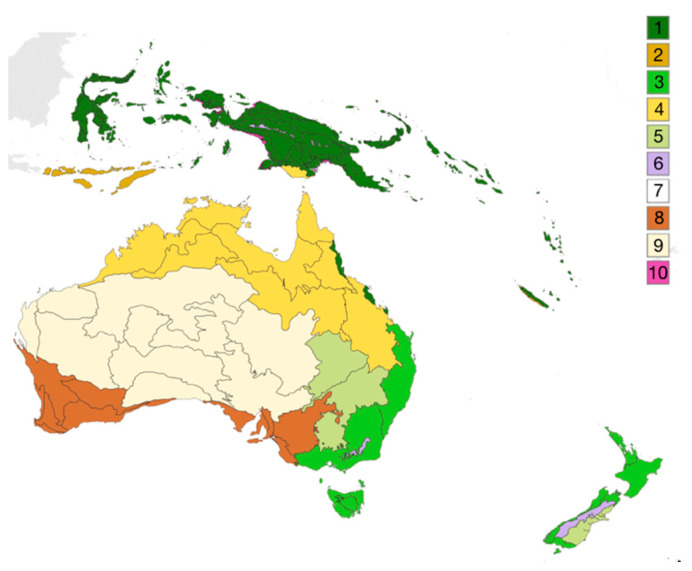
Map of the Australasian ecozone and biomes. Each color represents a biome of the Ecozone: (**1**) Tropical and subtropical moist broadleaf forests, (**2**) Tropical and subtropical dry broadleaf forests, (**3**) Temperate broadleaf and mixed forests, (**4**) Tropical and subtropical grasslands, savannas, and shrublands, (**5**) Temperate grasslands, savannas, and shrublands, (**6**) Montane grasslands and shrublands, (**7**) Tundra, (**8**) Mediterranean forests, woodlands, and shrub, (**9**) Deserts and xeric shrublands, (**10**) Mangrove. From [83].

To illustrate these concepts, the example of the Australasian ecozone is taken (Figure 3). This ecozone is composed of 10 biomes (Figure 3) and 82 ecoregions (not represented on the figure), among which the Australian “Kimberly tropical savanna” can be identified as an example.

Facing the definition of the ecological concepts above mentioned, it appears that ecozones are deeply linked with the historical evolution of geographically separated areas (mainly from the super-continent Gondwana dispersion) and ecoregions are intrinsically linked with a geographic component.

During this step of ESS identification, focusing on geographic boundaries or geological movements doesn’t seem to be particularly relevant. Thus, biomes are prescribed as an adequate level to perform a first association between environmental constraints and ESS. The interest of focusing on biomes is that they precisely synthesize the interaction between the environmental constraints and the associated species, which is the core of our strategy.

Moreover, since a given biotope can be associated with different biocenoses depending on the geographic location, considering an iterative phase of divergence based on geographic parameters can allow biomimetician to potentially access more specific ESS. Thus, where geographic areas appeared as a bias in the initial phase, using the ecological concept of biome as an initial step leads biomimetician to access the list of all the geographic areas presenting the association of biocenosis with a given biotope of interest: ecoregion.

**Figure 4:**
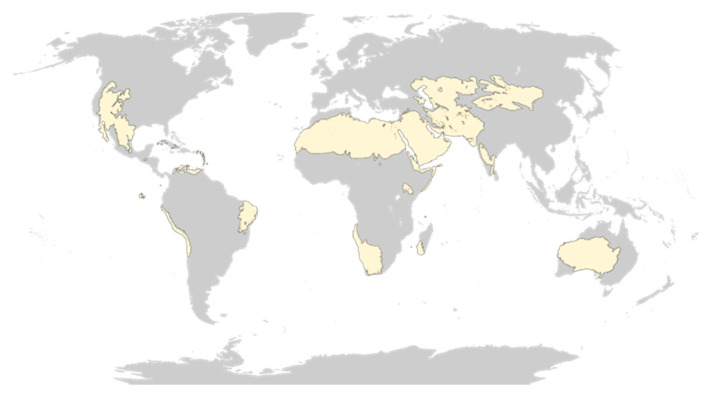
Map of the 99 ecoregions (in yellow) presenting a “Deserts and xeric shrublands” biome. From [84].

For example, the biome “deserts and xeric shrublands” corresponds to 99 ecoregions around the globe (Figure 4).

In this context, ESS are defined as the final environmental space chosen by the team to perform the search of biological models. Depending on the project, the resources or the expertise of the team, biomes, ecoregion, or even highly specific ecosystems can be considered as ESS.

### 3.2. Findability and the Identification of Biological Models of Interest

The 4th step of the biomimetic process is an exploration step aiming at searching for biological solutions to solve given abstracted problems. However, as previously presented, the 3rd step can lead to various type of output. Either the process is performed following the approach proposed in Section 3.1.2. leading to FSS or ESS, or the team doesn’t, and the inputs of the 4th step are only FSS, formalized as adequate requests for their various search tools. Either way, the idea is to keep on formalizing the biomimetician’s specific contributions.

#### 3.2.1. First Descriptive Step: Concepts Associated with the 4th Step

As it was the case for the 3rd step, the terminology can also appear quite ambiguous during the 4th step. Therefore, this section presents existing concept in phylogenetics and defines a few new concepts specific to biomimetics that will be used in the rest of the article.

As previously presented, organisms aren’t only linked by their abiotic constraints, and inter-organism relationships can also be a way to extend the search. We won’t deal with ecological relationships, even if we deeply encourage practitioners to consider it, since we already made our case on the interest of integrating ecology as a biological field of interest.

This section then rather focuses on another biological field, the study of the ancestral relatedness of groups of organisms, whether alive or extinct [85]: phylogenetics. Its main biological objectives and concepts are thus quickly presented before one of its potential application in biomimetics is detailed.

**Figure 5:**
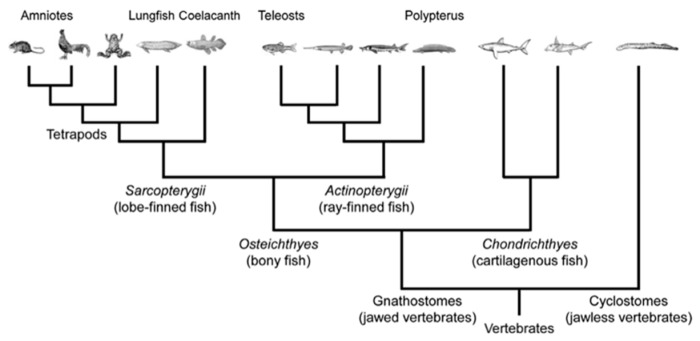
Example of a simplified phylogenetic tree of vertebrate. Adapted from Yamamoto 2017 [86].

In biology, the concept of phylogenetics refers to the study of species’ evolution and leads to their classification (taxonomy). Species evolutionary relationships are then represented through phylogenetic trees (Figure 5).

Before modern advances in genetics, classifications were mainly based on morphological or ecological traits. For example, the phylum *Mollusca* gathers soft (morphological trait) marine (ecological trait) invertebrate (morphological trait) organisms called mollusks. The advent of genetics led to an unmatched level of precision, allowing classifications not only based on given morphological or environmental traits, but on species’ genome.

**Figure 6:**
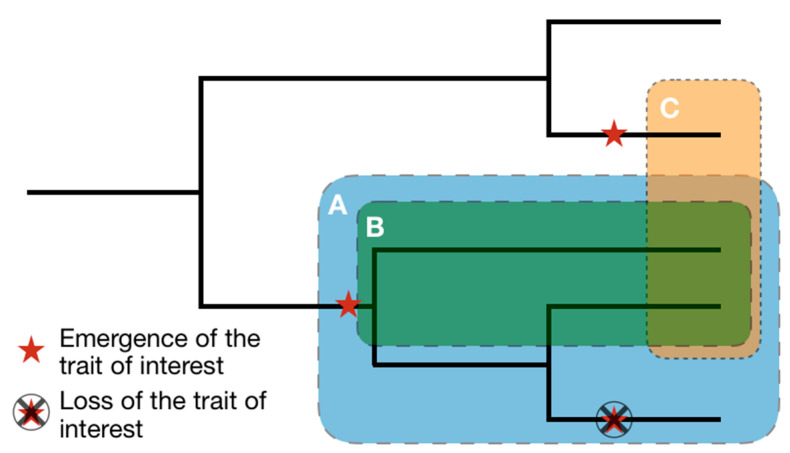
Illustration of the concepts of (A) monophyletic, (B) paraphyletic and (C) polyphyletic groups.

For biomimetician to extract and manipulate information made available in those trees, the concepts of mono, para and polyphyletic groups are illustrated (Figure 6) and their definitions are presented.

A monophyletic group is a cluster of species composed of a common ancestor and all its descendants, regardless of the evolutionary paths they followed past their common ancestor. The taxa composing a monophyletic group share synapomorphy, which are traits derived from their common ancestor.

A paraphyletic group is a cluster of species composed of a common ancestor and some of its descendants sharing common traits called a plesiomorphy. For example, if we study an ancestral trait A which was lost by some descendants, then the common ancestor and all its descendants still bearing the trait A will form a paraphyletic group with respect to those which have lost the trait. As a result, various paraphyletic groups can be defined within a monophyletic one, depending on the considered trait. The emergence, modification or loss of traits then represent the source of paraphyletic groups.

A polyphyletic group is a cluster of species which doesn’t share an immediate common ancestor. If these taxa share similitude of traits that don’t originate from a common ancestor, then these similarities are called homoplasies, or analogies if those traits are functions. They result from an independent evolutionary path: the convergent evolution process. For example, a bird and a butterfly both have wings leading to the function of flight, but their common ancestor doesn’t have wings and so both taxa have independently developed wings during their respective evolutionary process.

From a biomimetic standpoint, the 4th step is called identification of biological models, but the term model, even if it appears to refer to its analogical sense in the context of biomimetics, doesn’t specify by itself its biological nature.

To prevent any misunderstanding biological models are defined in this article as the specific biological construct that allows a given problem to be solved. For example, the lotus’ leaf surface represents a biological model. It protects the plant from bacterial and fungus and maintains its ability to harvest sunlight through super-hydrophobicity and self-cleaning properties.

In this context, we also defined the concept of a biological carrier as an organism carrying all or part of a biological model. In this context, organisms can appear as intermediate solutions.

In some cases, the biological carrier presents the whole biological model. For example, any desert organisms have a biological model for extreme heat resistance, but organisms only represent a first step towards the identification of these solutions.

In other cases, the biological carrier presents only part the biological model. The biological model is thus composed of a network of organisms and again identifying one organism appear as a first step towards the identification of the solutions.

Finally, a biological carrier can also be an equivalent to a biological model. For example, if the biological strategy appears to be of behavioral nature.

Based on these various concepts, the next sub-section presents the impact of phylogenetics on the 4th step of the biomimetic process.

#### 3.2.2. Prescriptive Step: From Solution Spaces to Biological Models

During the 4th step of the process, current tools face the impediment presented in the state of the art (Section 1.2.3). We propose other approaches through first the identification of biological carriers before identifying biological models in a second time. Thanks to biology-centered knowledge and know-how, a design team should then be able to complement the use of current tools and uncover whole new paths for models’ identification.

From an Ecological standpoint, numerous biodiversity databases are specialized in gathering the various species present in specific geographic areas. These databases can focus on specific taxon, like Avibase on birds [87], or specific region, like the biodiversity database of the Scientific Committee on Antarctic Research [88], or gather a wide range of species distributed worldwide like the database designed by the GBIF, Global Biodiversity Information Facility [89].Various functionalities, such as the interactive map on the GBIF database (gathering 6,586,578 species on the 29.02.20), can then allow biomimetician to identify biological carriers from ESS.

Phylogenetic tools then offer a way to use biological data as raw material in order to find more biological data through a cascading effect. Thus, from a single species identified using classic biomimetic tool, like existing databases, phylogenetic tool may lead to the identification of related groups of species. Indeed, a phylogenic tree presenting enough information may allow biomimetician to track the emergence of a trait of interest back to its source, i.e., a common ancestor, and identify its associated descendants.

As a result, taxa composing this group are susceptible to possess the trait of interest. Within this group, some species may have evolved, and the trait of interest may have mutated or even disappeared. Through their variability, these groups should present various versions of the original trait but adapted to different contexts. Even species which have lost the trait can be of interest since we can wonder why the trait disappeared and if it has been replaced by another structure, then representing another resolution strategy. Depending on the studied taxonomic level, the search will reveal close solutions as a result of shared evolutionary history (search focusing on close taxonomic groups such as shared genus) or different biological models with different solutions (search focusing on higher taxonomic level such as shared order) [63].

The use of biological tools and reasoning based on organisms then offers new ways of performing the 4th step of the process. It aims at allowing teams to reach a second phase of search amplification, exploiting biological data with biological tools to generate a new set of biological keywords and requests through an iterative method. Relevant keywords are selected and further deepen (divergence), where irrelevant keywords are dropped (convergence). Functional, ecological and phylogenetic solution spaces (gathering taxonomic groups of interest) can then be studied separately or combined to cross-reference the information and refine the search leading to multiple inferences as advised by Linsey [90] to increase the team’s ability to find analogical solutions.

**Figure 7:**
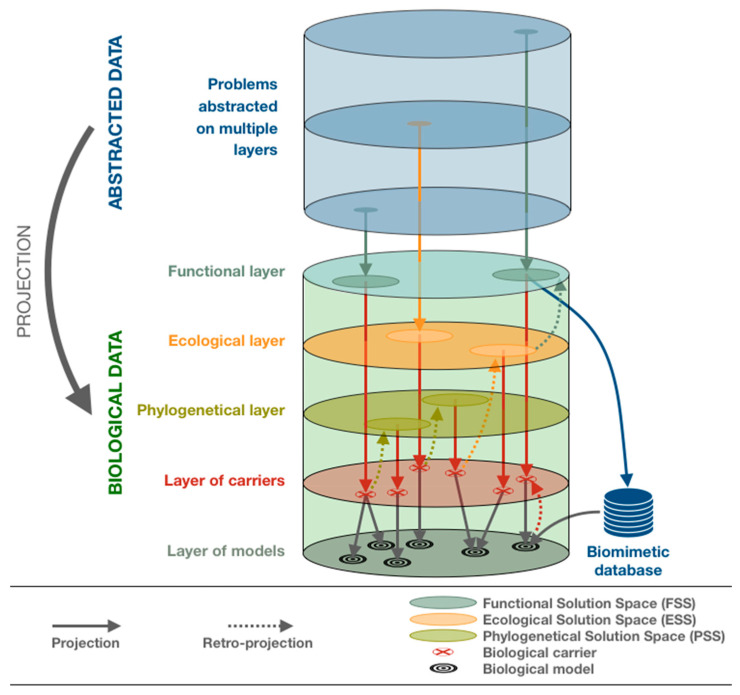
Search amplification through biological tools.

Thus, an initial FSS can lead to the identification of a first biological carrier A and associated model A. Based on ecological or phylogenetic biological reasoning, along with identified biological scientific papers, the ecosystems related to the biological carrier A, and its phylogenetic classification can be determined, leading biomimetic teams to perform an iteration based on those newly identified solution spaces. Since solution spaces are characterized by keywords, this new approach also appears to be compatible with most pre-existing tools based on keywords. Through the information gathered during the generation of solution spaces, iterations allow a biological contextualization of the reasoning and search. The search scope should be amplified and better defined at each iteration (Figure 7).

To illustrate this approach, we will consider the example of a design team searching for biological models targeting heat protection, having already identified a first organism of interest *Atta vollenweideri* through a functional request (FSS 1: Protect from temperature) in AskNature. This initial search led the team to consider the regulation of nest’s temperature through ventilation on the database page (https://asknature.org/strategy/turrets-ventilate-nest/). Considering *Atta vollenweideri* phylogenetic classification, the team can study high taxonomic groups like the Hymenoptera order, intermediary related groups like the Vespidae super-family (sharing the same infra-order) or closely related groups like the Myrmicinae sub-family, (https://www.ncbi.nlm.nih.gov/Taxonomy/Browser/wwwtax.cgi?mode=Info&id=592326). Based on the functional keywords associated with the initial organism (FSS 1.1: ventilation) and phylogenetic keywords, a new iteration of the search can be performed, this time on broader search engine like google scholar: “(Nest) Ventilation” AND “Myrmicinae” OR “Vespidae” OR “Hymenoptera”. In order to limit the search, we will only consider “easy identification” that we will characterize as the articles on the first page of results and having keywords related to the request in their title:

- When focusing on Myrmicinae, the search leads to studies on the genus *Atta*, and brings further information on these species’ ability to ventilate their nest based on the latest research findings [91,92] and associated studies of reference [93,94], as along as alternative solution by other species of the same genus, such as nest relocation under canopee coverage by *Atta Sexdens* [95]. These articles may also lead biomimetic team to identify other taxonomic groups sharing the same FSS, like the genus *Acromyrmex* [92] which is also part of the Myrmicinae sub-family or *Macrotermes,* in a genus of termites, part of a much higher taxonomic group, the Neoptera infra-class. As a result of their greater taxonomic distance, species of these groups, such as *Acromyrex heyeri* or *Macrotermes belicosus,* have developed different solution to the problem of thermal regulation: the adaptation of the nest’s thatch thickness and porosity [96].
- When focusing on Vespidae, the search led to no direct solutions.
- When focusing on Hymenoptera, the search leads to the identification of an additional article of interest [97]. Interestingly, it appears after a deeper analysis of the article that the keyword “ventilation” is link to the respiratory system and not directly with thermal regulation. However, along the article several ESS are detected (like the Mojave Desert or the Saharan desert) as part of the biome “Deserts and xeric shrublands” and associated with various species such as *Messor pergandei* or *Cataglyphis bicolor.* Article specifically studying these species are also identified, leading to the identification of a new model such as discontinuous ventilation cycles (DVC) to reduce water loss in *Cataglyphis bicolor* [98].

At the end of this iteration, new organisms have been identified, *Acromyrex heyeri, Macrotermes belicosus, Messor pergandei, Cataglyphis bicolor,* and their associated biological models underlined. Based on these results, the team can generate new PSS through the same phylogenetic tool, such as *Neoptera, Macrotermes, Messor, Cataglyphis,* etc. Associated with the divergence of PSS, the results also lead to a divergence in FSS, like with the identification of the keyword “thermoregulation” (FSS 1.2), and the identification of the ESS “deserts and xeric shrublands”. Altogether, these newly determined PSS, FSS and ESS will lead the team to generate alternative request and further increase the number of identified biological organisms and models during the next iteration.

Moreover, an PSS that appears unfruitful during one iteration, like “(nest) ventilation” AND “Vespidae” in the example, can become of interest associated with newly determined FSS. Thus, “thermoregulation” AND “Vespidae” leads to numerous models of interest, such as (8) a multiple layer nest or the use of external cavity as thermic protection, or (9) the transfer of heat to the nest by body conduction following thermal gradient [99].

Through such iterative reasoning, various types of carriers may be identified. We define these carriers as follow:

- Biological carriers of reference are organisms which, by themselves, represent biological models directly usable by the team. We suggest four cases that can lead to a biological carrier of reference. Organisms may (1) have already been used in previous biomimetic projects or described in biomimetic articles, (2) have been studied from an interdisciplinary standpoint, more specifically, when chemical, physical or mathematical studies have been performed on biological models, (3) have been extensively studied from a biological standpoint, as model organisms (*Mus musculus, Drosophila melanogaster, Escherichia coli*, etc.), as key organisms for a given field of application (*Homo sapiens* for medicine, *Bos taurus* for agriculture, etc.), as subjects of biological studies specifically focusing on the model of interest, or (4) be particularly well known by a teammate who then ensures the explanation of the associated biological model. Carriers of reference correspond to the final step toward the identification of biological models. Existing databases mostly gather biological carriers of reference, leading them to be the best known by the overall community. Based on the example previously presented, *Atta vollenweideri* and *Macrotermes belicosus* can be considered as carriers of reference because of (1) and (3) [99,100].
- Intermediary carriers are often firstly considered as potential carriers of reference before a lack of studies, a lack of resources or their inadequacy with the project requirement, make their associated models too hard to extract or irrelevant. Nevertheless, through literature search and functional, ecological, or phylogenetical tools, these carriers may be used as keywords to identify and better characterize solution spaces. Where biological carriers of reference can be used as intermediary carriers, the reverse is not true. Based on the example previously presented, *Cataglyphis bicolor* can be considered as an intermediary carrier as it can lead to the identification of other, better-studied, carriers, such as *Cataglyphis bombycina* [101].
- Carriers of diversification carry alternative versions of the biological models described in the carriers of reference. They might be less studied, farther from the technical context and so potentially harder to transfer, but they represent the biological diversity surrounding the model. Contrarily to intermediary carriers, their models are at least partly extracted, increasing the variability of a given biological model of interest. Based on the example previously presented, some representants of the *Atta* genus, such as *Atta sexdens,* can be considered as carriers of diversification with respect to *Atta vollenweideri*.

The definition of those three categories (Table 2) should help design teams during the 4th step by giving them the ability to classify the various types of concepts they face and help them prepare the 5th step by avoiding the confusion of the carriers’ types.

Facing the concepts described in this section, it must be underlined that the type of a given carrier can evolve depending on scientific progress and on the team’s skills or experience. As previously explained, these biological carriers are only intermediary answers. Once they are identified, the associated biological models need to be extracted from them. This extraction then relies on the biomimetician’s skills and the existence of scientific studies on the given biological carrier.

Where direct search for biological models was underlined as a difficulty in the state of the art, we support that the search for biological information targeting a given biological species in the scientific literature should be easier. Considering biological carriers as intermediary solution may also make easier the identification of researchers in biology which appear to be rather more specialized on given organisms than on biological models.

Moreover, since the source of information is represented by published biological articles, the amount of available biological models will evolve simultaneously with the biological fundamental research which represent one undeniable asset for the approach.

Such an amount of information then leads us to consider the precision and relevance parameters. The state of the art underlined that the difficulty of the 4th step isn’t only the limited amount of available information but also the relevance of the gathered information. The tools and approaches described in this article allow practitioners to characterize new solution spaces, supporting cross-referenced search and selection steps between a biological perspective and existing functional approaches.

On that aspect, existing studies, such as the work of Badarnah et al. [47] underlined in the state of the art, present a great framework to use. Increasing the amount of biological information to consider in pinnacles’ comparison matrix for example can be a way to approach the selection step.

More evaluations targeting biomimetic teams are being carried out to refine the method and identify additional good practices to consider. The main experimental difficulty so far is that biomimeticians don’t exist yet, and as a result we cannot fully evaluate the impact of such reasoning requiring knowledge and know-how in biology. On the other hand, through a retroactive reasoning, any advancements in the determination of the theoretical framework describing potential biological knowledge and know-how of interest leads us to better characterize the biomimetician.

### 3.3. Impact on Biomimetician’s Definition and Training

Facing biomimetics impediments identified in the state of the art, we argue for the integration of biological expertise in biomimetic design teams through a new profile that we referred to as the biomimetician in this article. However, such an integration appears possible only if some requirements are met. Without even considering the legitimacy of integrating biological expertise, that has been supported by various articles [7,8], a few questions emerged from the state of the art:

- Who are biomimeticians?
- How are they going to be integrated within biomimetic teams?
- What to expect from them?
- How are they going to meet these expectations and so what is their knowledge and know-how?

Facing the fact that those profiles are new, it is logical that engineering design studies only start to look at what it would mean to include this profile in terms of opportunities.

To that end, it appears that the more biological fields and tools are judged relevant for the practice of biomimetics, the better biomimeticians will be defined. In this context, this research article supports the characterization of biomimeticians by identifying two biological fields, ecology and phylogenetics, that appear relevant for the practice of biomimetics. More specifically, some concepts and tools have been presented as knowledge and know-how for biomimeticians to acquire and deepen during their academic education.

About their roles during the practice of biomimetic, this article described new ways of performing the 3rd and 4th steps of the biomimetic process, with the aim of making biomimeticians’ potential contributions as complementary as possible with the pre-existing teams’ practice. We argue that only a collaborative and interdisciplinary work will allow biomimetics to reach its full potential.

**Figure 8:**
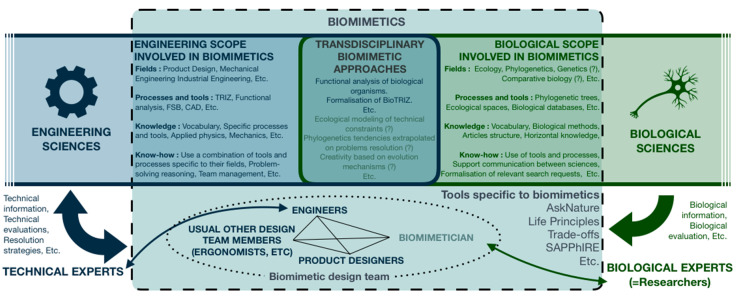
Diagram representing the updated summary of the identified fields, methods, tools and actors involved in biomimetics.

The formalization of biomimetician know-how and knowledge represents a true scientific challenge as biology encompasses numerous specific fields and communities. Figure 8 then models the contribution of this article on the definition of the biomimetician (Figure 8).

This work then only opens the door on the research axis representing the establishment of biomimeticians. Moreover, where this article focuses on their skills originating from biology, other studies will be needed to deal with biomimeticians’ skills originating from engineering or design sciences.

## 4. Discussion

The objective of this article is to initiate the integration of biological fields within biomimetics and the proper definition of biomimeticians. In this context, our findings aren’t final but represent one step towards the establishment of this new transdisciplinary stakeholder to be integrated into biomimetic teams.

To implement biological fields and practices, we narrowed down the study to the first two “biology-centered” steps of the biomimetic process, the projection of the faced problems into biology to identify solution spaces (step 3), and the search and identification of biological strategies (step 4).

**Figure 9:**
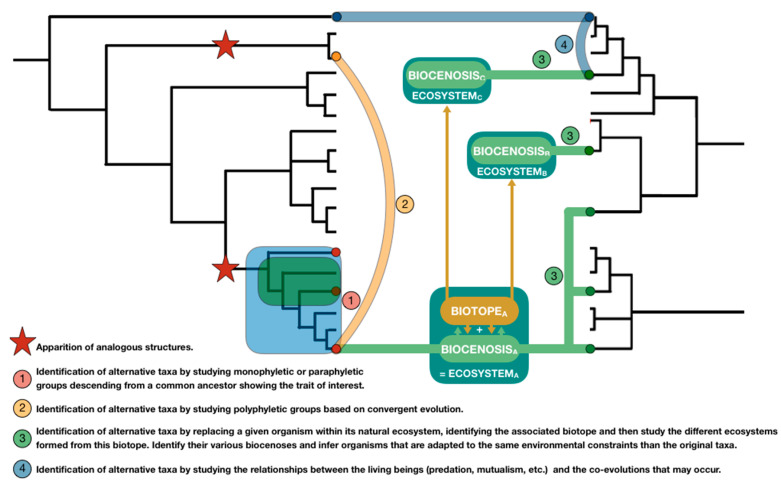
Synthesis of conceptual combinations between phylogenetic and ecological tools to identify organisms of interest.

First, by studying the conceptual aspects surrounding those biology-related steps we identified two fields of interest: ecology and phylogenetics. Those theoretical bridges offer a first set of biological tools to be used during the practice of biomimetics (problems projection, identification of environmental constraints, use of biomes and ecoregions, GBIF database, generation of relevant keywords, etc.). Figure 9 summarizes the various conceptual bridges exposed in this article (Figure 9).

Based on this exploratory work, various limitations on the use of reasonings and tools from ecology and phylogenetics must be underlined and can be associated with perspectives:

- We mainly studied abiotic environmental constraints (constraints that are not due to living beings), living aside ecological approaches investigating the interactions between biological systems (parasites and hosts, predators and preys, mutualistic symbiosis, etc.) which can also represent relevant identification pathways to explore.
- We assumed that biological data is always available in the literature, which isn’t the case. Biological classifications present species regardless of the amount of research performed after they were discovered. This potential lack of information appears as a strong impediment to anticipate. Tools that allow an initial screening of biological organisms based on their associated research effort could be a first step in addressing the problem.
- Since modern phylogenetic trees are mostly based on genetic distance without mentioning the implication on functional traits, new tools combining functional and ecological/phylogenetic classifications should represent a real step forward in their adaptation to biomimetic practices.
- One can argue that using taxonomic tools confines the search for models within a restricted branch of the tree of life. However, as they can bridge species based on their similarity of traits, and so on convergent evolution, polyphyletic groups should allow to expand solution spaces. Once a new species is identified, new mono or paraphyletic groups from other branches can then be described, expanding the searching area. Therefore, tools gathering studies on convergent evolution cross-referenced with functional, ecological, and phylogenetic criteria may be highly valuable.
- Tools from biology, whether they are ecological or phylogenetic tools, aren’t designed to be used in biomimetics. Hence, they aren’t optimized or ergonomics when used as biomimetic tools. A new generation of tools adapting those tools from biology to biomimetic practices should be of great interest.

In this article, we focused on two biological fields of interest because they appear easily understandable and highly relevant, but numerous other biological fields remain to be explored. These potential new strategies diverge from classic ones since they are conducted by team members trained in biology, using biological knowledge and know-how. To echo the ambiguous “biological tools” identified in the state of the art, the tools and concepts presented in these articles are truly biological since they are used daily by a part of the community of biologists.

So far, our study focused on the 3rd and 4th step of the biomimetic design process as they are the first to be linked with biology, but this logic of integration should be generalized. Considering biological fields and tools should lead us to take new types of inputs and outputs into account for the various steps, like the ESS, PSS and FSS in this article. Doing so, the process may need to be adapted to be able to properly integrate biological tools and practices.

Finally, in the absence of actual biomimeticians, our work remains mostly theoretical so far. Some examples and demonstration have been used to illustrate and show the potentialities of such reasoning, but these strategies need to be repeated numerous times on various subjects to be deepened and further adapted. These research axes represent a tremendous amount of work, but we believe that they also represent truly innovative and interdisciplinary ways of looking at the future of biomimetic methodological research.

## 5. Conclusions

Facing the current impediments in biomimetic practices, various papers have been supporting the integration within biomimetics design teams of an actor having a background in biology that we named “biomimetician” in this article. In this context, we formulated the following research question: what are biomimetician’s specific biological knowledge, know-how, and tools?

The hypothesis of this article was that phylogenetics and ecology could be part of biomimetician’s skills. Thus, we formalized conceptual and practical bridges between biomimetic design teams’ needs and these biological fields’ potential contributions. Then, we investigate their impacts on biomimetic practices, processes, and biomimetician’s training.

This study also allowed us to underline several limitations of current biological approaches in biomimetics. Hence, we preconized various research axes and guidelines to design a new generation of tools and methods combining a biological standpoint while fitting biomimetic needs and characterizing biomimetician.

The final objective of our work is the deep integration of biology during biomimetic practices in order to overcome obstacles currently identified in the literature and support biomimetics’ spreading. We defend the idea that such a transition will be promoted by getting out of the paradoxical situation where biomimetics is defined as an interdisciplinary approach but where biology is only considered for its data. We argue that biomimetic practices should also be based on biological know-how. Through the definition of biomimeticians, as transdisciplinary actors trained in biology and specialized in biomimetics, we want to structure the integration of biological fields that can be identified relevant for biomimetics.

In that respect, this article represents a first step towards this objective and initiate the identification and operational integration of biological fields.

## Figures and Tables

**Table 1:** Comparison of the solving approaches identified in the state of the art.

Current Solutions	Advantages	Drawbacks	References
Databases & associated approaches	Functionally structured information Pre-identified content Ergonomics & ease of use	Low number of models Unbalanced number of models depending on the functional fields or the kingdom of life	[29,32,37,38]
Natural language web search (AI is still under development)	Based on the total amount of published biological information Very precise and scientific content can be found Already daily used tools (Google, etc.)	Difficulty of designing relevant requests Ill-structured data Very unprecise and erroneous content can be found A strong sorting step is required	[6,33,39,40,41]
Approaches based on highly abstracted principles	Bridge engineering and biology Ergonomics & ease of findability by changing the reasoning scale (highly abstracted solutions)	Difficulty of formulation of an abstracted request Difficulty of using raw abstracted solutions to generate technological innovations Potential fixations	[24,35,36,42,43]

**Table 2:** Type of carriers and characteristics

Type of Carriers	Amount of Data	Use during the Process	Input for
**Intermediary**	Low	Identify solution spaces	Step 4
**Reference**	High	Extract biological models of reference	Step 4 & 5
**Diversification**	Intermediate	Supplement the models of reference	Step 4 & 5
